# HIV patients with latent tuberculosis living in a low-endemic country do not develop active disease during a 2 year follow-up; a Norwegian prospective multicenter study

**DOI:** 10.1186/s12879-014-0667-0

**Published:** 2014-12-17

**Authors:** Nadine Durema Pullar, Harald Steinum, Johan Nikolai Bruun, Anne Ma Dyrhol-Riise

**Affiliations:** Department of Internal Medicine, Section for Infectious Diseases, University Hospital of Northern Norway, Tromsø, N-9038 Norway; Department of Clinical Medicine, Faculty of Health Sciences, University of Tromsø, Tromsø, N-9037 Norway; Department of Infectious Diseases, Trondheim University Hospital, Trondheim, N-7004 Norway; Department of Clinical Science, Faculty of Medicine and Dentistry, University of Bergen, Bergen, N-5021 Norway; Present address: Department of Infectious Diseases, Oslo University Hospital (Ullevål), pb 4956, Nydalen, Oslo, 0424 Norway; Institute of Clinical Medicine, University of Oslo, Oslo, 0316 Norway

**Keywords:** Tuberculosis, HIV, IGRA, QuantiFERON-TB, Tuberculin skin test, Norway, TB low-endemic, Preventive therapy, Follow-up

## Abstract

**Background:**

Interferon-γ release assays (IGRA) serve as immunodiagnostics of tuberculosis (TB) infection to identify individuals with latent TB infection (LTBI) eligible for preventive anti-TB therapy. In this longitudinal study of HIV-infected LTBI patients we have observed for possible progression to active TB as well as evaluated repeated IGRA testing in a TB low-endemic setting.

**Methods:**

QuantiFERON TB-Gold In-tube*®* assay (QFT), TB-SPOT.*TB®* (TSPOT) and tuberculin skin test (TST) were performed on 298 HIV-patients recruited from seven out-patient clinics in Norway. Patients with active TB, LTBI and negative IGRA were followed with repeat QFTs and clinical evaluation over a period of 24 months.

**Results:**

Seven HIV-patients (median CD4 count 270; IQR 50–340) were diagnosed with active TB at inclusion, all IGRA positive. Sixty-four (21%) HIV-patients (median CD4 count 471; IQR 342–638) were diagnosed with LTBI and of these 39 (61%) received TB preventive treatment. Neither treated nor untreated HIV-infected LTBI patients developed active TB during the 24 months. At baseline, the median interferon-γ (INF-γ) level measured by QFT was 3.48 IU/ml (IQR 0.94 – 8.91 IU/ml) for treated LTBI compared to 1.13 IU/ml (IQR 0.47 – 4.25 IU/ml) for untreated LTBI patients (p = 0.029). The QFT reversion rates were 75% for active TB, 23% for treated LTBI and 44% for untreated LTBI, whereas the conversion rate for the non-TB group was 7% despite no new TB exposure. There was no significant difference in the trend of INF-γ levels over time between treated and untreated LTBI patients.

**Conclusion:**

The prevalence of LTBI is high among HIV-patients, but the risk of developing active TB seems to be low in patients with high CD4 counts in this TB low-endemic setting. In several patients, especially with baseline IFN-γ levels close to cut-offs, the QFT tests reverted to negative independent of preventive anti-TB treatment indicating possibly false positive tests. This highlights the importance of defining reliable cut-offs for immunodiagnostic tests and deferring preventive therapy in selected patients. Randomized studies with longer follow-up time are needed to identify HIV-patients that would benefit from LTBI treatment in a TB low-endemic setting.

**Electronic supplementary material:**

The online version of this article (doi:10.1186/s12879-014-0667-0) contains supplementary material, which is available to authorized users.

## Background

For the diagnosis of latent tuberculosis infection (LTBI) the tuberculin skin test (TST) has been replaced or supplemented by interferon-gamma (IFN-γ) release assays (IGRA) in many tuberculosis (TB) low-endemic countries due to their simplicity and improved specificity [1]-[4]. However, there are limitations to the use of both IGRAs and TST in the presence of Human Immunodeficiency virus (HIV) infection as both tests can be negative due to T-cell anergy in patients with low CD4 counts [5]-[8]. Furthermore, neither IGRA nor TST can distinguish between latent and active TB, the tests may be negative in active TB patients or remain positive in patients previously treated for TB infection [7]-[9]. Recent reports have also highlighted the intra-assay variability and conclude that positive QuantiFERON-TB Gold In-Tube tests (QFT) with INF-γ levels less than 0.59 IU/ml should be interpreted cautiously [10]. An alternative cut-off of 0.70 IU IFN-γ/ml has also been suggested [11]. The predictive value of TST has been documented by several studies both in TB-endemic and low-endemic countries [12]. Although, Diel *et al.* report two year progression rates of 8-15% for TB among IGRA-positive patients, data are limited for IGRAs as prognostic markers in HIV-infected patients [13].

In Norway, 80-90% of TB cases and 60-70% of new HIV cases occur in immigrants predominantly from TB-endemic countries [14],[15]. Still, there are no surveillance data on HIV/TB co-infections due to confidentiality considerations prohibiting identifiable registration of HIV status. In a survey from the period 2005–2010 (unpublished), only 2-7% of active TB and 1-5% of LTBI patients were confirmed HIV-infected, but 40% and 50% of active TB and LTBI patients, respectively had not been HIV tested. Harstad *et al’s* study of asylum-seekers in Norway revealed poor follow-up routines for persons at high risk for TB where only 1% of LTBI cases received preventive treatment [16].

We have previously reported a prevalence of 26% for a positive IGRA test from a cross-sectional study of a cohort of HIV positive patients [17]. Origin from a TB-endemic country, prior active TB and TB exposure were risk factors for positive IGRA. Interestingly, our findings indirectly support the hypothesis of waning of latent TB infection in a setting of low infectious burden even in HIV patients, as IGRA prevalence was lower in patients with more than 10 years stay in Norway. We now report prospective data on the same HIV-infected cohort where we have observed for possible progression to active TB over two years in two comparable groups of HIV patients with and without preventive anti-TB treatment. We have also compared reversions and conversions rates for repetitive QFT tests in the same time-period. Further, we have studied fluctuations of QFT results over time in patients previously treated for active TB as well as in HIV-infected without TB infection.

## Methods

### Study participants

HIV-infected patients were recruited to the study as previously described [17]. Briefly, background information on HIV infection and risk factors for TB were collected on HIV positive individuals over the age of 18 years recruited from seven out-patient infectious disease clinics in Norway between January 2009 and October 2010. Information on previous active TB diagnosed and treated outside of Norway was based on self-report. Study participants were followed for two years with QFT and routine blood samples including CD4 counts. At inclusion, all patients were clinically examined for active TB, a chest X-ray was performed and a sputum sample obtained for acid fast staining and culture. Those with a positive IGRA or suspected TB infection were further assessed for active TB with an additional induced sputum sample or bronchoalveolar lavage (BAL). In selected patients organ specific scans and biopsies were obtained according to clinical judgement. Patients were questioned about travel to TB-endemic countries, possible re-exposure for TB and new symptoms of active TB between testing points.

A written informed consent was obtained from each participant. The study was approved by the Regional Committee for Medical and Health Research Ethics (REK-Vest) and by the Norwegian Social Science Data Services (NSD).

### Interferon-gamma Release Assays and Tuberculin Skin Test

The QuantiFERON TB-Gold In-tube*®* assay (QFT) (Cellestis Ltd, Qiagen, Chadstone, VIC, Australia) was performed at baseline and repeated at 3, 6, 12 and 24 months after initiation of therapy in LTBI and active TB patients and after 12 and 24 months in HIV patients with no signs of present TB infection. INF-γ levels ≥ 0.35 international units (IU)/ml (TB antigen response minus nil response) were considered a positive test. Reversion of a QFT test was defined as a positive QFT test at baseline that became negative (INF-γ < 0.35 IU/ml) at one of the subsequent tests, whereas conversions of a QFT test was defined as a change from baseline negative to positive QFT at one of the subsequent test points specified.

T-SPOT.*TB®* (TSPOT), performed according to manufacturer instructions (Oxford Immunotec Ltd., Abingdon, UK) was taken in parallel to QFT only at inclusion in patients attending one of the participating hospitals and was not repeated during follow-up. Positive results were defined as > 7 spot forming units.

A TST by the Mantoux method was administered once at study start after the IGRA test was taken to avoid the possibility of boosting and read after 72 hours (0.1 ml tuberculin PPD RT23 2 TU, SSI, Copenhagen, DK) [18]. Induration ≥5 mm was considered a positive test according to guidelines for HIV-infected persons [19].

### Definitions of study groups

*Active TB diagnosis* was given when the presence of *M. tuberculosis* in a clinical sample was laboratory confirmed by acid-fast bacilli on microscopy, by growth in culture or by a nucleic acid amplification test. A clinical case of active TB was defined according to the following criteria: a positive TST or IGRA and/or signs and symptoms compatible with TB (abnormal chest radiograph or imaging study, or clinical evidence of current disease) and a completed diagnostic evaluation followed by initiation of efficient treatment with standard anti-TB therapy [20]. *LTBI diagnosis* at baseline was given to patients with a positive IGRA (QFT and/or TSPOT) and no indication of current or previous active TB. Participants with a prior active TB, which could explain a positive IGRA and with no signs or symptoms of active TB at baseline, were classified as *prior active TB.* Finally, those that had received preventive TB therapy before entering the study were defined as *prior LTBI.* Patients with a negative baseline IGRA and no previous or current TB infection were classified as *no TB*. Preventive treatment for LTBI, consisting of either 6 months of isoniazid or 3 months of rifampicin and isoniazid, was decided and implemented by the responsible clinician according to national guidelines [2].

### Statistical analysis

All analyses were performed using STATA 12 software (STATA Corporation, College Station, Texas, USA). Descriptive data are presented as median (interquartile range (IQR)), mean or numbers (percentage). Univariate odds ratios (OR) for risk factors associated with positive IGRA and TST in patients with prior TB were obtained by logistic regression models. Quantitative QFT trends over time for LTBI, active TB and prior TB patients were analyzed using linear mixed models (LMM) with random intercept. Wilcoxon rank sum test was used to compare PPVs (estimated by “The Online TST/IGRA interpreter”) between patient groups.

## Results

### Diagnosis of tuberculosis infection by IGRA in HIV patients

**Figure 1:**
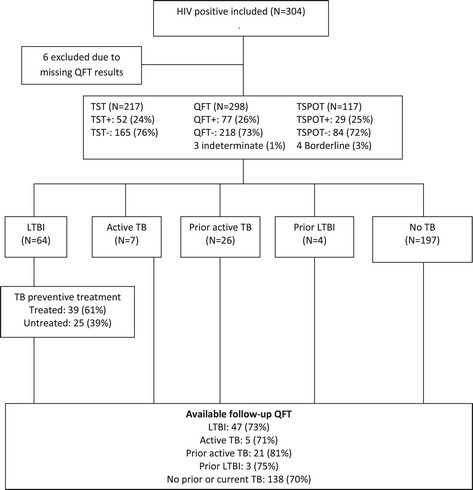
**Study design and study groups.** QFT = QuantiFERON-TB Gold. TSPOT = T-SPOT.*TB*. TST = Tuberculin skin test. TB = Tuberculosis. LTBI = Latent TB infection

A total of 304 HIV-positive patients were enrolled in the study, however QFT results were missing in six persons and these were excluded from the final analysis. The remaining 298 were classified as *active TB* (N = 7), *LTBI* (N = 64), *prior active TB* (N = 26), *prior LTBI* (N = 4) and *no TB* (N = 197) (Figure 1). Characteristics of the various HIV groups stratified by origin from TB-endemic versus low-endemic regions are given in Table 1. Seventy-nine patients did not show up for administration of the TST or for reading after 72 hours. The fractions of positive IGRAs in the various TST strata are shown in Table 2.

**Table 1:** **Characteristics of HIV-patients according to origin and stages of tuberculosis infection**

	TB-endemic countries (N = 217)					TB low-endemic countries (N = 81)		
	LTBI	Prior TB*	Active TB	Prior LTBI	No TB	LTBI	Prior TB	No TB
	(N = 60)	(N = 23)	(N = 7)	(N = 4)	(N = 123)	(N = 4)	(N = 3)	(N = 74)
**Age**								
**Median (IQR)**	37 (31–41)	41 (36–46)	43 (39–48)	45 (37–49)	36 (31–42)	44 (40–47)	50 (43–60)	53 (46–60)
**Gender**								
**Female N (%)**	36 (60)	11 (48)	3 (43)	2 (50)	87 (71)	2 (50)	1 (67)	19 (26)
**Male N (%)**	24 (40)	12 (52)	4 (57)	2 (50)	36 (29)	2 (50)	2 (33)	55 (74)
**Years of stay in Norway**								
**Median (IQR)**	5 (2–8)	7 (3–11)	5 (1–6)	4 (3–5)	7 (4–10)	n/a	n/a	n/a
**Years of HIV infection**								
**Median (IQR)**	3 (2–7)	8 (3–10)	7 (3–10)	6 (5–8)	6 (3–9)	16 (9–20)	6 (3–23)	6 (3–11)
**Nadir CD4 cells/μl**								
**Median (IQR)**	270 (170–488)	80 (40–190)	190 (50–290)	92 (81–190)	188 (87–290)	135 (90–180)	101 (100–230)	192 (90–354)
**CD4 cells/μl at enrolment**								
**Median (IQR)**	472 (342–638)	340 (240–430)	270 (50–340)	541 (442–608)	408 (276–590)	465 (375–595)	320 (310–560)	468 (312–690)
**ART at enrolment N (%)**	28 (47)	17 (74)	5 (71)	4 (100)	88 (71)	4 (100)	3 (100)	49 (66)
**Exposure to TB**	16 (27)	1 (4)	2 (29)	2 (50)	8 (6)	0	1 (33)	6 (8)
**TST positive (%)**	30 (60)^1^	8 (47)^3^	1 (25)^5^	0^7^	10 (10)^8^	0^10^	1 (50)^12^	2 (5)^14^
**TST mm (median-IQR)**	10 (0–15)	3 (0–15)	0 (0–5)	0 (0–0)	0	0 (0–1)	11 (0–22)	0
**QFT positive (%)**	56 (93)	9 (35)	6 (86)	1 (25)	0	4 (100)	1 (33)	0
**QFT IU/ml (median-IQR)**	2.00 (0.56–6.20)	0.34 (0.34–1.88)	6.33 (0.81–10)	10 (10–10)	0.34	0.91 (0.49–5.63)	0.34 (0.34–10)	0.34
**TSPOT positive (%)**	20 (87)^2^	6 (50)^4^	3 (60)^6^	….	0^9^	0^11^	0^13^	0^15^
**TST/QFT discordance N (%)**	22 (44)	5 (29)	2 (50)	0	10 (10)	3 (60)	0	2 (5)
**TST-/QFT+**	19	2	2	0	0	3	0	0
**TST+/QFT-**	3	3	0	0	10	0	0	2

TB: tuberculosis. LTBI: latent TB infection. ART: antiretroviral therapy. QFT: QuantiFERON TB Gold. TSPOT: T-SPOT.*TB*. n/a: not applicable. *Excludes 2 diagnosed with LTBI and 4 diagnosed with current active TB. ^1^TST available in 50 LTBI pasients from TB-endemic countries. ^2^TSPOT available in 23 LTBI pasients from TB-endemic countries. ^3^TST available in 17 patients with prior TB from TB-endemic countries. ^4^TSPOT available in 12 patients with prior TB from TB-endemic countries. ^5^ TST results available in 4 patients with active TB. ^6^ TSPOT results available in 5 patients with active TB. ^7^TST results available in 2 patients with prior LTBI. ^8^ TST available in 96 “no TB” patients from TB-endemic countries. ^9^TSPOT available in 44 “no TB” patients from TB-endemic countries. ^10^TST available in 3 LTBI pasients from TB low-endemic countries. ^11^TSPOT available in 2 LTBI pasients from TB low-endemic countries. ^12^TST available in 2 patients with prior TB from TB low-endemic countries. ^13^ TSPOT available in 2 patients with prior TB from TB low-endemic countries. ^14^TST available in 43 “no TB” patients from TB low-endemic countries. ^15^TSPOT available in 28 “no TB” patients from TB low-endemic countries.

**Table 2:** **Comparison between TST and IGRA results**

	TST (N = 74)	TSPOT+ (N = 23)	TST (N = 217)	QFT+ (N = 60)	INF -γ (IU/ml) Median (range)
TST (mm)		No. (% of TST)		No. (% of TST)	
**<5**	56	10 (18)	165	26 (16)	0.81 (0.39-10)
**5-10**	7	6 (86)	17	8 (47)	7.20 (1.44-10)
**11-14**	3	1 (33)	12	10 (83)	2.60 (0.57-10)
**≥ 15**	8	6 (75)	23	16 (70)	4.68 (0.5-10)

TST = Tuberculin skin test. IGRA = Interferon-gamma Release Assay. TSPOT = T-SPOT.*TB*. QFT = QuantiFERON-TB Gold. INF-γ = Interferon-gamma .TST results were missing for 63 negative QFTs, 17 positive QFTs and 1 indeterminate QFT. TST results were missing for 6 positive TSPOTs, 36 negative TSPOTs and 1 borderline TSPOT.

### Longitudinal QFT testing during treatment of active tuberculosis in HIV patients

Seven HIV patients were diagnosed with active TB at enrolment, five with pulmonary TB and two with extra-pulmonary TB, all with origin from a TB-endemic country (Table 3). Five patients met laboratory definitions whereas two had clinical indications of pulmonary TB. Four had been previously treated for active TB abroad with a median time since end of treatment of 1.5 years, and one had developed a multidrug-resistant TB. Induced sputum for acid fast staining and culture was performed in all participants to identify active TB diagnosis in patients possibly missed by the lack of symptoms, a negative IGRA or negative chest X-rays, but this diagnostic approach did not reveal additional cases of TB.

**Table 3:** **Demographics and clinical data for HIV patients diagnosed with active TB**

Origin	Age	Gender	Years in Norway	Previous TB (years)	Symptoms	CD4 count	HIV RNA (copies/ml)	TST (mm)	QFT (IU/ml)	TSPOT	Culture/PCR positive	Chest x-ray pathology	TB localization	Resistance
South-east Asia	40	F	6	n/a	Yes	302	<20	nd	2.66	nd	+/+	-	Pulmonary	-
East-africa	45	M	6	n/a	Yes	340	8750	10	10	+	+/+	-	Abdominal	-
Central-africa	33	F	5	1	Yes	190	70	nd	10	nd	+/+	-	Lymph node	MDR
Central-africa	43	M	3	2	No	480	<20	0	0.62	b.l	-/nd	+	Pulmonary	n/a
East-africa	42	F	1	1	No	50	>100000	0	0.34	+	-/nd	+	Pulmonary	n/a
East-africa	50	M	1	11	No	50	>100000	0	0.81	-	+/nd	-	Pulmonary	-
West-africa	38	M	7	n/a	No	270	>100000	nd	10	+	+/+	+	Pulmonary	INH

n/a = noe applicable, nd = not done, b.l = borderline, MDR = multi drug-resistant TB, INH – isoniazide.

All active TB patients tested one or both IGRAs positive (Tables 1 and 3). Four of the five active TB patients with available follow-up QFT results were positive at enrolment, but only one remained positive after 24 months. There was a significant reduction of QFT levels already at 6 months (coeff.-3.10 IU/ml, p = 0.020) and further at 12 months (coeff. -2.59 IU/ml, p = 0.012). The patient with negative QFT and positive TSPOT at baseline maintained QFT negative results at all test points. There was no significant association between CD4 counts and QFT level trends over time in active TB patients.

### Longitudinal QFT testing during treatment and follow-up of latent tuberculosis in HIV patients

Sixty-two patients in this cohort were diagnosed with LTBI according to study definition. Additionally, two patients with negative QFT and missing TSPOT were tested during contact tracing of a contagious TB patient and based on the clinician judgement considered possibly infected and given preventive TB therapy (TST 0 and 10 mm) (Table 1). Within 6 months of diagnosis, 39 (61%) LTBI patients had started preventive TB treatment, the majority (92%) with rifampicin and isoniazid for 3 months. Median INF-γ value at baseline for treated LTBI was 3.48 IU/ml (IQR 0.94 – 8.91 IU/ml) compared to 1.13 IU/ml (IQR 0.47 – 4.25 IU/ml) for untreated LTBI patients (p = 0.029). Neither treated nor untreated LTBI patients developed active TB during the observation period of 24 months. However, five patients were lost to follow-up. No participants reported new exposure to an active TB case during the study period.

**Figure 2:**
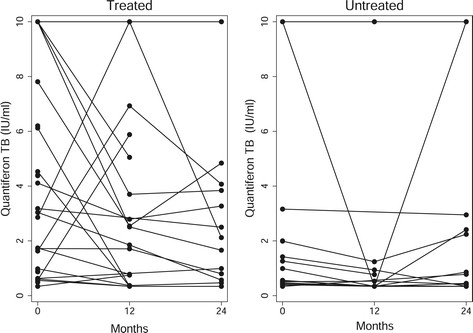
**Quantitative QFT responses over time in HIV patients with LTBI.** QFT responses (IU/ml) at baseline (0), 12 months and 24 months follow-up in LTBI patients receiving preventive TB treatment and in untreated LTBI. There was no significant difference in QFT responses over time between treated and untreated LTBI patients. QFT = QuantiFERON-TB Gold.

One or more longitudinal follow-up QFT was available for 47 of the 64 LTBI patients (Figure 2). For those patients with QFT available at both 12 and 24 months (n = 33), consistently positive QFT was seen in 12/17 treated LTBI patients (7 TST+, 2 TST-, 3 missing TST), 1/17 reverted to negative QFT already at 3 months (TST-), whereas 4/17 reverted at 12 months (3 TST-, 1 TST+), though one converted back to positive QFT at 24 months (TST+). In the untreated LTBI group, consistently positive QFT was seen in 4/16 patients (3 TST+, 1 missing TST), 7/16 reverted and stayed QFT negative during 24 months of follow-up (4 TST-, 3 missing TST) whereas 4/16 experienced QFT reversions, but became positive again at 24 months (3 TST-, 1 TST+). In addition, 1/16 patient with negative QFT at baseline became positive at 3 months (TST+, TSPOT+).

**Figure 3:**
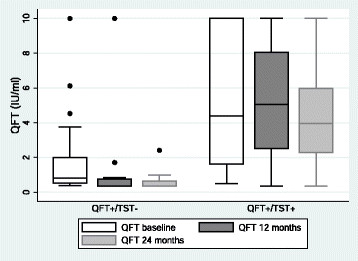
**Longitudinal QFT responses in HIV patients with positive and negative TST.** Boxplot showing median (line in box), interquartal range (box), upper and lower range excluding outliers (whiskers) and outliers (dots) of QFT responses at baseline, 12 and 24 months in patients with positive QFT and discordant and concordant TST results. Patients with positive QFT and positive TST had higher INF-γ responses at baseline and follow-up compared to patients with positive QFT and negative TST. QFT = QuantiFERON-TB Gold. TST = Tuberculin skin test.

Baseline IFN-γ levels were generally lower for untreated LTBI patients that reverted compared to those that remained QFT positive at 24 months but this was not significant (0.56 IU/ml versus 1.49 IU/ml, p > 0.05). There was no significant difference in the trend of INF-γ levels (IU/ml) over time between treated and untreated LTBI patients (Figure 2). In linear mixed models (LMM) analyses LTBI patients with a positive TST had higher INF-γ results over time compared to TST negative patients (coeff 2.887 IU/ml, p < 0.001), irrespective of preventive therapy (Figure 3). Higher nadir CD4 counts were also associated with slightly higher INF-γ over time (coeff. 0.004 IU/ml per cell/μl, p = 0.024). Travel to TB-endemic countries between QFT testing points had no significant effect on INF-γ trends over time. The four LTBI patients from TB low-endemic countries were not given treatment. In three of these the QFT test reverted to negative, and the fourth patient experienced a reduction in INF-γ levels during follow-up.

Applying an IFN-γ cut-off of 0.70 IU/ml at baseline, as has been suggested [13], 21 of the HIV-patients diagnosed with LTBI in our cohort would have tested QFT negative. However, ten (48%) had other indicators of LTBI such as recent TB exposure or a positive TSPOT. Among the untreated LTBI patients with baseline IFN-γ < 0.70 IU/ml, seven of the nine with available follow-up results maintained IFN-γ values < 0.70 IU/ml.

### Longitudinal QFT testing in HIV-patients previously treated for active tuberculosis

Thirty-two patients had been treated for active TB before enrolment in the study. Two patients were considered re-infected based on reported new TB exposure before entering the study and were thus classified as LTBI and 4 were diagnosed with new active TB. Ten of the 26 (38%) “*prior active TB”* patients tested QFT positive at baseline (Table 1). Among the patients with available follow-up QFT, 9/21 patients stayed QFT positive (5 TST+, 2 TST-, 2 missing TST), 9/21 patients with negative baseline QFT remained negative (5 TST-, 1 TST+, 3 missing TST) and 3/21 converted from QFT negative to positive after 12 months (2 TST+, 1 TST-), although one reverted to negative again at 24 months (TST-). There was no indication of new TB exposure in any of these patients.

In the univariate analysis high CD4 counts at inclusion was the only factor associated with persistently positive QFTs in patients with prior active TB (OR 14.05, 95% CI 1.57-125.55, p = 0.018) and in LMM analysis IFN-γ levels increased with increasing CD4 counts (coeff. 0.008 IU/ml per cell/μl, p = 0.003). There was no significant association between time since treatment and QFT results. Mean INF-γ values over time were significantly lower in patients with a previous pulmonary TB (mean 1.42 IU/ml, SD 1.97) compared to patients with extra-pulmonary TB (mean 3.99, SD 4.29, p = 0.02).

### Longitudinal QFT testing in HIV-patients with negative baseline IGRA

Follow-up QFT was available in 138 of the197 HIV patients classified as *no TB*. Conversion rates to positive QFT in this group were 7% at 12 months and 4% at 24 months. However, no patients reported exposure to TB during the study period.

## Discussion

This study describes the challenges of LTBI diagnosis and decision-making regarding preventive TB treatment in HIV patients in a TB low-endemic country as Norway. The prevalence of LTBI in our HIV positive cohort was high with the majority of patients originating from TB-endemic countries, in line with other studies [5],[7],[13],[21],[22]. Still, in our study, with over half of the HIV-patients on ART, none of the LTBI patients developed active TB during the 24 months of observation time regardless of preventive anti-TB treatment.

The lack of a diagnostic gold standard for LTBI leaves much up to clinicians to decide if the patients should receive preventive anti-TB therapy. In this study 94% of LTBI patients received the diagnosis based on a positive IGRA and 6% on clinical judgement. However, only 61% of the HIV-infected LTBI patients received preventive anti-TB treatment, although strongly advised by Norwegian and international guidelines [2],[4]. Poor implementation of anti-TB preventive treatment is also revealed in the study among asylum seekers by Harstad *et al.,* where none of the seven LTBI patients with HIV-infection were treated [16],[23].

According to a Cochrane review, LTBI treatment reduces the risk of active TB by 32% in HIV patients [24]. A Swiss HIV Cohort Study reinforced the importance of preventive treatment in a TB low-endemic setting [12]. In our study, clinicians refrained from treating the few QFT positive patients from TB low-endemic countries with no known TB exposure. The majority of these patients tested negative when QFT was repeated supporting a study of US-born HIV patients at low risk for TB in which 80% with initial positive QFT reverted to negative when retested after a median time of 40 days indicating false positive primary tests [25]. Also we observed a high QFT reversion rate of 44% in the untreated LTBI group. Further, untreated LTBI patients had lower IFN-γ values compared to treated patients in our study, indicating a tendency for clinicians to make decisions based on IFN-γ values. Fluctuations of IFN-γ values close to the cut-off of ≥ 0.35 IU/ml in the QFT assay are described in several studies supporting suggestions to adjust IFN-γ cut-off levels [10],[11],[18],[26] and define a borderline interval, for example 0.35 – 0.70 IU/ml [27]. Thus, we advocate a policy of retesting HIV patients with low INF-γ levels as well as to consider deferring preventive treatment in patients expected to have low risk of progression to active TB infection due to efficient ART [28].

The consequences of not treating LTBI in HIV infection are difficult to ascertain. Whereas there is an abundance of studies on the risk of progression to active TB in TST positive patients, data are more limited for IGRA positive patients [13]. We have not found data to support that higher IFN-γ levels in HIV infection increases the risk of developing active TB. The review by Diel *et al.* concludes that the positive predictive value (PPV) for progression to active TB of 3-14% with positive IGRA is higher than for TST [13]. Still, whereas low CD4 counts are associated with an increased risk of developing TB, the risk is less among HIV patients receiving efficient ART [12]. Our study where none developed active TB regardless of preventive therapy is in contrast to the Swiss study were 6.5% of HIV-patients not treated for LTBI acquired active TB during the follow-up of 52 months [12]. A PPV of 8-10% for progress to active TB infection in IGRA positive HIV infected patients within a two years period has also been documented from other TB low-endemic countries [29],[30]. The fact that CD4 counts were lower and fewer patients were on ART in these studies compared to our study could partly explain these conflicting data. Furthermore, patients with active TB might have been misclassified as LTBI, whereas active TB patients were carefully identified at inclusion in our study.

Tools such as “The online TST/IGRA interpreter” to assist diagnosis and treatment decisions aim to identify patients at risk for TB infection [31],[32]. Using this algorithm we calculated PPVs for TB infection of 93.2% - 99.9% for patients classified as LTBI in our cohort with the lowest PPV calculated for patients from TB low-endemic countries. HIV-infected with positive TST, negative IGRA and no history of TB exposure had PPVs for TB infection up to 95% by this calculator, whereas these patients were not diagnosed with LTBI in our study, none developed TB during the two years of follow-up and repeat QFT results were consistently negative. The calculated annual (7.4-8%) as well as the overall risk (100%) for active TB was high for HIV-infected patients. However, the algorithm does not include ART, CD4 count or negative TSTs making the algorithm less useful in a HIV positive cohort.

We observed that participants with concordant QFT and TST results at enrolment maintained higher IFN-γ levels throughout the observation time of 24 months [33],[34]. This may indicate a stronger and more stable immune response, which may have prognostic significance. In a recent study conducted in Taiwan, HIV patients with concordant positive TST and TSPOT results had a 7.8-fold increased risk of developing active TB if left untreated, suggesting that dual testing is more reliable [35]. Still, it is of note, for screening purposes TST administration and reading proved to be difficult in our study with 27% of participants failing to comply. It is nevertheless apparent from our data that the majority of IGRA positive HIV-patients do not progress to active TB. Prognostic biomarkers to identify those who will progress to active TB are however lacking. Thus, longitudinal studies of longer duration are needed to assess TB risk in HIV patients on efficient ART and high CD4 count.

Our study from a TB low-endemic setting where only 61% received preventive treatment made it possible to analyze fluctuations in QFT over time and compare treated and untreated IGRA positive patients as no patients reported TB re-exposure during follow-up. There was no significant difference in QFT over time in treated or untreated LTBI patients and travel to TB-endemic countries did not have an impact on QFT results. Higher nadir CD4 counts but not CD4 count during follow-up were significantly associated with higher IFN-γ levels in repeated QFT and reflect the effect of immune suppression upon IFN-γ responses. There was a significant reduction in IFN-γ levels during active TB treatment, but too few active TB patients to conclude. Other studies have also observed a decline in IFN-γ levels during TB treatment, but most studies conclude that QFT and TSPOT are unreliable biomarkers of treatment efficacy [36]-[40].

Four of the seven patients diagnosed with new active TB in our study were previously treated for active TB, including one patient that developed multidrug-resistant TB. This illustrates the importance of full clinical evaluation in HIV patients with a prior active TB, particularly if the prior treatment and follow-up is unknown. Nevertheless, the fact that IGRAs may stay positive in patients who have previously been adequately treated and cured for active TB is a major obstacle to the use of IGRA to monitor treatment efficacy [5],[7],[23]. In our study 38% of patients previously treated for TB had positive QFT. Interestingly, higher CD4 counts was the only factor associated with persistently positive QFT which may reflect higher numbers of circulating TB-antigen specific effector T-cells. Furthermore, higher INF-γ values over time were observed in patients with previous extra-pulmonary TB compared to pulmonary TB, suggesting that a stronger T-cell response is retained after successful treatment in this group [41].

The main limitation of our study is the inadequate QFT retesting due to logistic problems that limits evaluation of QFT dynamics over time. Furthermore, the low number of active TB cases precludes adequate analysis of test performances during therapy in this patient group.

## Conclusion

IGRAs seem to be the most useful screening test for LTBI in HIV infection in our TB low-endemic clinical setting as the TST was hampered by poor attendance and 16-18% of IGRA positive patients tested TST negative. The LTBI prevalence among HIV patients from TB-endemic countries was high. Still, LTBI treatment implementation was low as HIV patients with low baseline IFN-γ results were generally not given treatment. Neither treated nor untreated HIV patients with LTBI, the majority on ART, developed active TB during the two years of observation time. In both groups there were reversions and conversions of QFT tests and QFT proved to be unreliable in monitoring LTBI treatment efficacy. Fluctuations of INF-γ levels close to the established cut-offs for the QFT-test also justify that retesting and follow-up without preventive treatment is appropriate for selected HIV patients. High CD4 count and positive TST was associated with higher IFN-γ over time and may indicate persistence of strong immune responses, which may have prognostic implications. Still, randomized longitudinal studies are needed to adequately identify HIV patients that would benefit from LTBI treatment in a TB low-endemic setting.

## Authors’ original submitted files for images

Below are the links to the authors’ original submitted files for images. Authors’ original file for figure 1 Authors’ original file for figure 2 Authors’ original file for figure 3
