# Supplementary figures and images for: HIV patients with latent tuberculosis living in a low-endemic country do not develop active disease during a 2 year follow-up; a Norwegian prospective multicenter study
Source: BMC Infect Dis. 2014 Dec 17;14:667. doi: 10.1186/s12879-014-0667-0 (PMC4273430; doi:10.1186/s12879-014-0667-0)

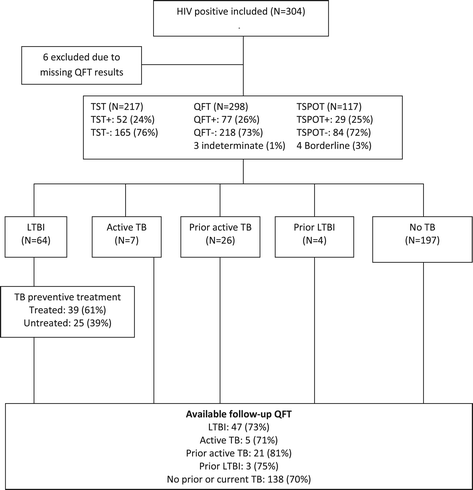

Supplement: Supplementary file 1 — Authors’ original file for figure 1 [file 12879_2014_667_MOESM1_ESM.gif]

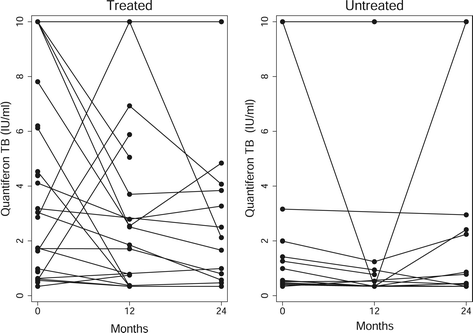

Supplement: Supplementary file 2 — Authors’ original file for figure 2 [file 12879_2014_667_MOESM2_ESM.gif]

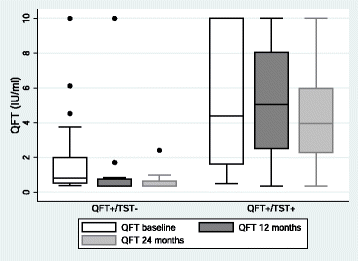

Supplement: Supplementary file 3 — Authors’ original file for figure 3 [file 12879_2014_667_MOESM3_ESM.gif]
